# Viral Etiological Agent(s) of Respiratory Tract Infections in Symptomatic Individuals during the Second Wave of COVID-19 Pandemic: A Single Drive-Thru Mobile Collection Site Study

**DOI:** 10.3390/pathogens11040475

**Published:** 2022-04-15

**Authors:** Aleksandra Kozinska, Karolina Wegrzynska, Magdalena Komiazyk, Jaroslaw Walory, Izabela Wasko, Anna Baraniak

**Affiliations:** Department of Biomedical Research, National Medicines Institute, 00-725 Warsaw, Poland; a.kozinska@nil.gov.pl (A.K.); k.wegrzynska@nil.gov.pl (K.W.); m.komiazyk@nil.gov.pl (M.K.); j.walory@nil.gov.pl (J.W.); i.wasko@nil.gov.pl (I.W.)

**Keywords:** COVID-19, SARS-CoV-2, variants of SARS-CoV-2, respiratory viruses, co-infection

## Abstract

One of the tools to contain the SARS-CoV-2 pandemic was to increase the number of performed tests and to improve the access to diagnostics. To this effect, mobile collection sites (MCSs) were established. This study was performed on samples collected at the MCS between November 2020 and March 2021. We aimed to confirm/exclude SARS-CoV-2, differentiate SARS-CoV-2 variants, and detect other respiratory pathogens. SARS-CoV-2 and other respiratory viruses were identified by RT-qPCRs. A total of 876 (46.35%) SARS-CoV-2 positive specimens in the diagnostic tests were identified. The wild-type variant was determined in 667 (76.14%) samples; the remaining 209 (23.86%) samples specimens were identified as Alpha variant. A total of 51 (5.6%) non-SARS-CoV-2 cases were detected in retrospective studies. These accounted for 33 cases of mono-infection including rhinovirus (RV), human adenovirus (HAdV), human metapneumovirus (HMPV), enterovirus (EV), and influenza virus, and 18 cases of co-infection (SARS-CoV-2 with RV or HAdV or HMPV, and RV with EV). Our research shows that the results obtained from the MCS have value in epidemiological studies, reflecting national trends on a micro scale. Although the spread of COVID-19 is a major public health concern, SARS-CoV-2 is not the only pathogen responsible for respiratory infections.

## 1. Introduction

Respiratory tract infections (RTIs) are the most common infections in patients who present themselves for medical consultation and constitute a major source of morbidity and mortality worldwide, accounting for approximately 3 to 5 million deaths annually [1,2]. RTIs cause a range of infections limited to the upper respiratory tract (rhinitis, sinusitis, pharyngitis, or tracheitis) and/or the lower respiratory tract (mainly bronchitis and pneumonia). The etiological factors of these infections may be various microorganisms including viruses, bacteria, and fungi [1,2,3]. Viruses are responsible for the majority (nearly 80%) of acute RTIs and until recently, the most common viruses were influenza, human respiratory syncytial virus, parainfluenza, and human adenovirus [2]. Over time, rhinoviruses and coronaviruses were discovered, and as methods for their detection improved, their importance in viral respiratory diseases was recognized. They are considered to be responsible for 50% to 75% of upper respiratory tract infections [4]. The most common bacterial pathogens in upper and lower respiratory tract infections are *Streptococcus pneumoniae*, *Haemophilus influenzae*, *Moraxella catarrhalis*, *Mycoplasma pneumoniae*, *Chlamydophila pneumoniae*, and *Legionella pneumophila*. Fungal RTIs are a major clinical problem, especially in immunocompromised patients, and are mainly caused by *Aspergillus*, *Cryptococcus*, and *Pneumocystis* [5].

In December 2019, Chinese health authorities detected several cases of unusual severe pneumonia in Wuhan that were caused by a novel coronavirus. This virus has been called severe acute respiratory syndrome coronavirus 2 (SARS-CoV-2) and is responsible for respiratory tract infection, coronavirus disease 2019 (COVID-19) [6,7]. SARS-CoV-2 has spread worldwide, and COVID-19 was granted pandemic status by the World Health Organization (WHO) in March 2020 [8]. COVID-19 can range from asymptomatic or mild symptoms that can be easily missed in the early stages of the disease, to severe or even critical, with mortality [9,10,11,12,13]. The most common mild clinical manifestations are fever, cough, myalgia, headache, and dyspnea; anosmia and diarrhea are less common. The diagnosis of COVID-19 remains a challenge because the typical clinical symptoms of this disease are indistinguishable from those caused by other respiratory viruses, which makes difficult to choose the correct therapy [9,10,11]. In addition, individuals with SARS-CoV-2 may have co-infection with other respiratory pathogens, which can complicate the diagnosis and treatment of COVID-19 [14,15,16,17,18,19]. Over time, there has been an increasing number of publications describing viral co-infections with SARS-CoV-2. However, as this is a newly discovered virus, further studies are needed to determine whether co-infections cause increased disease severity, mortality, shock, or the need for assisted ventilation. Detection of such interactions is crucial to design treatment strategies and determine their epidemiological impact [20]. Therefore, identification of the causative respiratory pathogens is of great importance for management and containment of the epidemic (in a specific location or population, e.g., in the family or at work) and also pandemic (worldwide) spread of SARS-CoV-2, and also contributes to reducing the isolation time of patients, especially those infected only with other common respiratory viruses. It also results in reduced hospital admissions (free beds for severe cases) and a faster return to community life, with implications for health service efficiency, health system expenditure, and the country’s economy.

Several methods of detecting etiological agents of RTIs are available. The most commonly used are rapid direct antigen tests, direct testing of fluorescent antibodies or culture. However, looking at test sensitivity, specificity, identification time, and the scope of pathogen detection, nucleic acid amplification tests seem to be the best method [21]. The gold standard for SARS-CoV-2 detection is real time quantitative polymerase chain reaction (RT-qPCR) [22]. This method can also be used to detect other respiratory pathogens, including bacteria and fungi.

There are various ways of preventing the spread of COVID-19, e.g., keeping social distance, using protective masks (especially in closed rooms), washing hands, and using disinfectants. One of the tools to contain the SARS-CoV-2 pandemic was to increase the number of performed tests and to improve the access to diagnostics. To this effect, mobile collection sites (MCSs) were established. MCS is the station (most often a tent or an ambulance) where material is collected for testing. Such a solution allows for screening a large number of people in a short time, which enables safe contact between the patient and the medical care worker. MCSs can also serve as vaccination centers or pharmacies. They are organized mainly at hospitals and clinics, but also in places ensuring convenient access by public transport or by car (city stadiums, parking, and squares). Launching the MCS requires little financial and organizational commitment and brings many benefits such as relieving emergency departments (eliminating the need for the patient to come to the hospital for a test, securing against deplete personal protective equipment, and other hospital resources) [23].

The aim of the study was analysis of samples suspected to be SARS-CoV-2 positive, sent to the National Medicines Institute (NMI) from one of the MCS in Mazovia. The study concerned (i) confirmation/exclusion of SARS-CoV-2, (ii) differentiation of SARS-CoV-2 variants in positive samples to analyze changing epidemiology, and (iii) detection of other respiratory pathogens to evaluate co-infections among tested cases. The study was case-control (retrospective) and showed the relationship between exposure (a harmful factor) and its effect (a disease phenomenon).

## 2. Materials and Methods

### 2.1. Specimen Collection

The clinical samples collected from individuals with suspected COVID-19 between November 2020 and March 2021 were analyzed. The specimens were not subject to selection; all patients were qualified for diagnostic study. The samples originated from a drive-thru MCS in Ostrow Mazowiecka and were sent to the NMI in Warsaw together with basic patient demographic data obtained through a laboratory form. The patients completed a laboratory form giving consent to the collection of material for testing and General Data Protection Regulation (GDPR) consent. All samples were nasopharyngeal cavity swabs transported in virus-dedicated media (all containing Hanks’ balanced salt solution) of different producers (Biocomma Limited, Shenzen, China; Liofilchelm, Roseto degli Abruzzi, Italy; and ClinicScience, Nanterre, France). The collected specimens were assigned codes that prevented identification of personal information at the time of testing. After the diagnostic testing for SARS-CoV-2, both positive and negative specimens were stored at −80 °C, pending further studies.

### 2.2. Nucleic Acids Isolation and RT-qPCR Tests

The nucleic acids were extracted using the NucleoMag Pathogen kit (Machery-Nagel, Duren, Germany) according to the manufacturer’s instruction. A manual procedure was applied with magnetic blocks, as described previously [22]. RT-qPCRs were carried out according to the program recommended by the test manufacturers (Section 2.2.1, Section 2.2.2 and Section 2.2.3) using the Applied Biosystems QuantStudio 6 Pro Real-Time PCR System (Life Technologies Holdings Pte Ltd., Singapore). The results were interpreted based on the quantification cycle value according to manufacturers’ recommendations.

#### 2.2.1. Detection of SARS-CoV-2

Identification of SARS-CoV-2 for diagnostic testing was performed using the commercial MutaPLEX^®^ Coronavirus (SARS-CoV-2) kit (Immundiagnostik AG, Bensheim, Germany) which detects 3 viral genes: SARS-CoV-2–specific S and RdRP genes, and E gene that is characteristic for both known SARS viruses. Re-detection of SARS-CoV-2 verifying quality of deposited specimens after thawing for retrospective studies was carried out using the MutaPLEX^®^ RespiraScreen 1 kit (Immundiagnostik AG, Bensheim, Germany) which identifies only the E gene.

#### 2.2.2. Differentiation of SARS-CoV-2 Variants

All SARS-CoV-2 positive samples were tested for differentiation of the most common variants present at the time of collection using two assays: the Bosphore^®^ SARS-CoV-2 Variant Detection Kit v1 (Anatolia Geneworks, Istanbul, Turkey), which identifies lineage B.1.1.7 (Alpha variant), and the ID™ SARS-CoV-2/VOC evolution Pentaplex (ID Solutions, Grabels, France), which detects lineage B.1.351 (Beta variant), lineage B.1.617.1 (Kappa variant) and lineage B.1.617.2 (Delta variant).

#### 2.2.3. Identification of Other Respiratory Pathogens

The retrospective detection of other respiratory pathogens was performed using the previously mentioned the MutaPLEX^®^ RespiraScreen 1 kit, which identifies influenza A/B viruses (FLUV), and human respiratory syncytial virus A and B (HRSV), in addition to SARS-CoV-2. Furthermore, the samples were tested with the Bosphore^®^ Respiratory Viral Basic Panel Kit (Anatolia Geneworks, Istanbul, Turkey), which detects viral and bacterial causative agents of respiratory infections, including parainfluenza 1/2/3/4 viruses (HPIV), human adenovirus (HAdV), enterovirus (EV), rhinovirus (RV), human metapneumovirus (HMPV), and *M. pneumoniae* and *L. pneumophila*, respectively.

### 2.3. Statistical Analysis

The statistical analysis was performed with STATISTICA Software (Version 9.0, StatSoft Inc., Tulsa, OK, USA). The statistical significance of differences between groups was determined by the two-sided chi-square test. A *p*-value <0.05 was considered statistically significant. The figures were created using GraphPad Prism 7.0 software (GraphPad Software, Inc., San Diego, CA, USA).

## 3. Results

### 3.1. Study Specimen Characteristics

A total of 1890 clinical samples were collected between November 2020 and March 2021: *n* = 806, *n* = 413, *n* = 281, *n* = 123, and *n* = 267 per month, respectively. The demographics are shown in Table 1.

**Figure 1:**
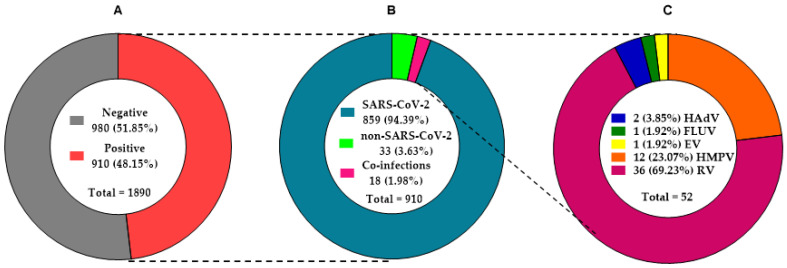
The frequency of the participation of viral agents in RTIs. (**A**) All clinical samples: negative and positive cases. (**B**) All positive cases: mono-infections (SARS-CoV-2 and non-SARS-CoV-2) and viral co-infections. (**C**) Non-SARS-CoV-2 viruses (51 cases of mono- and co-infections caused by 52 viruses, including HAdV—human adenovirus, FLUV—influenza virus, EV—enterovirus, HMPV—human metapneumovirus, and RV—rhinovirus).

The studied cohort consisted of 1041 female (55.07%) and 849 male (44.92%), ranging in age from 0 to 99 years, with median age of 51 years. The most common group were individuals aged 36–64 (*n* = 1023; 54.13%), followed by those aged ≥65 (*n* = 422; 22.33%), 19–35 (*n* = 342; 18.09%), 6–18 (*n* = 63; 3.33%), and 0–5 (*n* = 40; 2.12%). All patients reported symptoms of RTIs (general; they were not specified in the laboratory form) or contact with a person diagnosed with COVID-19. The viral agent(s) of respiratory tract infection was identified in almost half of the subjects (*n* = 910; 48.15%) (Table 1, Figure 1). Among them were 486 women (46.69% of all tested female, 53.41% of viral positive) and 424 men (49.94% of all tested male, 46.59% of viral positive). The most frequent viral positive cases were identified in the 19–35 (*n* = 175/342; 51.17%) and 36–64 (*n* = 518/1023; 50.64%) age groups, followed by those aged ≥65 (*n* = 179/422; 42.42%), 6–18 (*n* = 27/63; 42.86%), and 0–5 (*n* = 11/40; 27.5%).

### 3.2. Distribution of SARS-CoV-2

**Figure 2:**
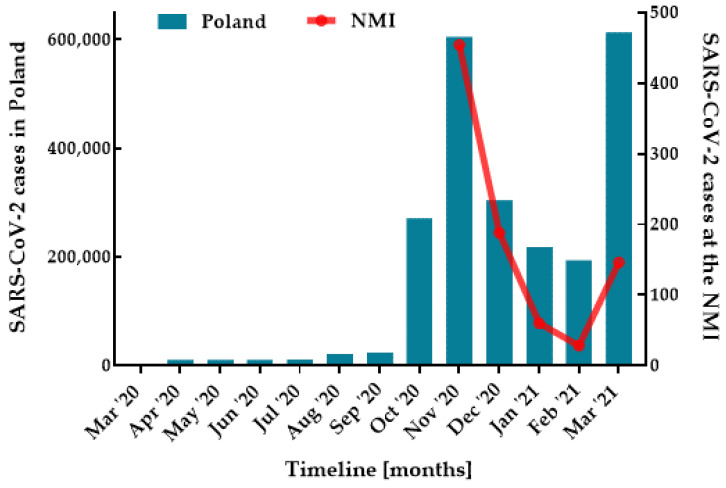
The monthly distribution of SARS-CoV-2 cases confirmed at the NMI (red line) against national data (blue columns).

The results of the SARS-CoV-2 detection are shown in Table 1 and Figure 1 and Figure 2. A total of 876/1890 (46.35%) SARS-CoV-2 positive specimens in the diagnostic tests were identified, which was subsequently 100% confirmed by retrospective studies. This represented 96.26% (*n* = 876/910) of all detected positive cases. The prevalence of SARS-CoV-2 varied with *n* = 454/806 (56.33%), *n* = 188/413 (45.52%), *n* = 60/281 (21.35%), *n* = 28/123 (22.76%), and *n*= 146/267 (54.68%) per month, respectively (Figure 2).

The SARS-CoV-2 mono-infection was detected in 859 samples (45.45% of all tested, 94.40% of viral positive cases, and 98.06% of SARS-CoV-2 positive). The study group consisted of 454 female (52.85% of SARS-CoV-2 positive) and 405 male (47.15% of SARS-CoV-2 positive) (*p* = 0.1722). The most frequent cases were identified in the 36–64 age group with *n* = 491 (48.0% of all tested and 94.79% of viral positive in this age group) and 19–35 with *n* = 164 (47.95% of all tested and 93.71% of viral positive in this age group), followed by those aged ≥65 with *n* = 173 (41.0% of all tested and 96.65% of viral positive in this age group), 6–18 with *n* = 24 (38.1% of all tested and 88.89% of viral positive in this age group), and 0–5 with *n* = 7 (17.5% of all tested and 63.64% of viral positive in this age group). The co-infection of SARS-CoV-2 with other respiratory pathogens was found in 17 specimens (0.9% of all tested, 1.87% of viral positive, and 1.94% of SARS-CoV-2 positive).

### 3.3. Changing Epidemiology of SARS-CoV-2

**Figure 3:**
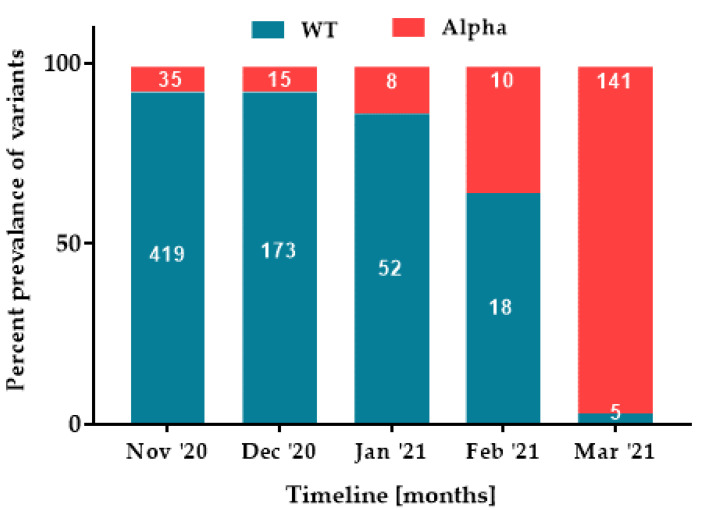
The percentage prevalence of SARS-CoV-2 variants. The blue color represents the wild-type (WT) virus, the red represents the Alpha variant. The values in the columns indicate the number of samples.

All 876 SARS-CoV-2 positive samples were screened for differential Alpha, Beta, Kappa, and Delta variants. In general, the wild-type (WT) variant, was determined in 667 (76.14%) samples. The remaining 209 (23.86%) positive samples were identified as Alpha variant. The distribution of variants changed over the time in preference to the Alpha variant with *n* = 35/454 (7.71%), *n* = 15/188 (7.99%), *n* = 8/60 (13.33%), *n* = 10/28 (35.71%), and *n* = 141/146 (96.58%) per month, respectively (Figure 3).

### 3.4. Distribution of Non-SARS-CoV-2 Respiratory Viruses

Only viruses were detected in the tested samples; *M. pneumoniae* and *L. pneumophila* were not identified within the specimens. The most common identified virus was RV (*n* = 36/52; 69.23%), followed by HMPV (*n* = 12; 23.08%), HAdV (*n* = 2; 3.85%), EV (*n* = 1; 1.92%), and FLUV (*n* = 1; 1.92%). These represented 5.6% (*n* = 51/910) of all detected positive cases. The results of finding other respiratory viruses (including RV—rhinovirus, HAdV—human adenovirus, HMPV—human metapneumovirus, EV—enterovirus, and FLUV—influenza virus) are shown in Table 1 and Table 2, and Figure 1.

A total of 51 (2.7% of all tested, 5.6% of viral positive) non-SARS-CoV-2 cases (52 respiratory viruses) in the retrospective studies were identified. These accounted for 33 cases of mono-infection and 18 cases of co-infection (17 with SARS-CoV-2 and 1 with other respiratory viruses). There was no difference in the incidence of mono-infections and co-infections non-SARS-CoV-2 (*p* = 0.0369). The percentage 3.63% (*n* = 33) of positive non-SARS-CoV-2 viral mono-infection cases compared to 94.79% (*n* = 859) SARS-CoV-2 mono-infection and 0.11% (*n* = 1) vs. 1.87% (*n* = 17) for co-infection was significantly lower, *p* = 0.0002). The patients of non-SARS-CoV-2 mono-infections consisted of 20 female (60.61% of non-SARS-CoV-2 positive) and 13 male (39.4% of non-SARS-CoV-2 positive). The difference in the number of mono-infected individuals between the genders was not statistically significant (*p* = 0.3186). The most frequent mono-infection cases were identified in the 36–64 age group with *n* = 17 (1.37% of all tested and 3.28% of viral positive in this age group) and 19–35 with *n* = 9 (1.37% of all tested and 3.28% of viral positive in this age group), followed by those aged ≥65 with *n* = 4 (0.95% of all tested and 2.24% of viral positive in this age group), 6–18 with *n* = 2 (3.17% of all tested and 7.41% of viral positive in this age group), and 0–5 with *n* = 2 (5.0% of all tested and 18.18% of viral positive in this age group). The most common found virus responsible for mono-infections was RV (*n* = 22/33; 66.67%) identified in every age group (*n* = 2, *n* = 2, *n* = 6, *n* = 11, and *n* = 1, respectively) and each month (*n* = 3, *n* = 8, *n* = 5, *n* = 5, and *n* = 1 per month, respectively), followed by HMPV (*n* = 10/33; 30.30%) in patients aged 36–64 (*n* = 5) from December to March (*n* = 4, *n* = 1, *n* = 2, and *n* = 3, respectively), 19–35 (*n* = 3 in December), and ≥65 (*n* = 2) one each in January and February, and FLUV (*n* = 1/33; 3.03%) in January from woman belonged to 36–64 age group.

### 3.5. Co-Infection Rates

The results of the co-infection distribution (except the month of virus identification) are shown in Table 1 and Table 2 and Figure 1. Eighteen co-infections were identified among the studied cases, which constituted 0.95% of all tested and 1.98% of positive cases. A positive result for SARS-CoV-2 co-infection with other respiratory viral agent(s), as mentioned previously, was found in 17 samples (0.9% of all tested, 1.87% of viral positive, and 1.94% of SARS-CoV-2 positive). The most frequent cases were identified in the 36–64 age group with *n* = 10 (0.98% of all tested and 1.93% of viral positive in this age group), followed by those aged 19–35 with *n* = 2 (0.58% of all tested and 1.14% of viral positive in this age group), ≥65 with *n* = 2 (0.47% of all tested and 1.12% of viral positive in this age group), 0–5 with *n* = 2 (5.0% of all tested and 18.2% of viral positive in this age group), and 6–18 with *n* = 1 (1.59% of all tested and 3.7% of viral positive in this age group). The highest rate of SARS-CoV-2 co-infections was observed with RV (*n* = 13; nine males and four females) identified in each month except January (*n* = 8, *n* = 2, *n* = 1, and *n* = 2 per month, respectively) and every age group except 6–18. Two SARS-CoV-2 co-infections with HAdV (males in age groups: 6–18 and 36–64) in December and HMPV (female and male in 36–64 age group) in March and December were found. In addition, one RV and EV co-infection was detected in March at female over 65 years old.

## 4. Discussion

Since the first identification of SARS-CoV-2 in late 2019, the WHO has recorded more than 410 million confirmed cases of COVID-19, including almost 6 million deaths [24]. The COVID-19 pandemic spread worldwide causing health, economic, and social distresses. It represents not only a serious diagnostic/therapeutic problem, but also a major epidemiology and public health challenge, straining the resources of healthcare systems. To contain the pandemic, it is crucial to monitor and detect every infected person in order to apply the isolation and treatment. Therefore, the rapidity and accessibility of SARS-CoV-2 diagnostics is of great importance. The establishment of drive-thru MCSs was intended to relieve the burden on health-care facilities and increase the availability of detect virus tests. This testing strategy has improved and provided direct and easy access to diagnostic testing to reach the broader population. The drive-thru testing formats have previously been promoted as a safe and effective method for large volume testing initiatives. It allows direct detection the pathogen during pandemic situations and has the benefit of reducing the number of infectious persons entering and contaminating healthcare establishments as well as promoting social distancing [25]. Our research shows that the results obtained from the drive-thru are also of value in epidemiological studies, reflecting national trends on a micro scale.

In Poland, the first SARS-CoV-2 infection was registered on 4 March 2020, and by 14 February 2022, nearly 5.5 million people had contracted the disease and almost 110,000 people had died [26]. The present study includes clinical specimens from patients with suspected COVID-19 who presented for testing during the second wave of the pandemic at a drive-thru mobile collection point located in Mazovia. Most epidemiological studies on COVID-19 to date have rarely focused on samples taken from individuals at MCSs; the majority of investigations have involved hospital patients whose parameters could be followed during hospitalization [25,27,28,29]. The number of SARS-CoV-2 positive cases identified in the NMI reflected a nationwide trend [26], with a peak in November, then a decline until February and another increase in March (Figure 2), which was covered by the global data [24]. A total of 876 SARS-CoV-2 positive specimens in the diagnostic tests were identified. This corresponded to 859 mono-infections and 17 co-infections. SARS-CoV-2 was statistically significantly (*p* < 0.0001) more frequently responsible for respiratory tract infections than other viruses, which was in line with national data [30,31]. No gender differences in virus infection were observed; SARS-CoV-2 was detected in 466 (51.21%) females and 410 (45.05%) males. The majority (96.12%) of cases were identified in adults, that was statistically significant (*p* < 0.0001). Our observations on the population data are similar to those of other studies [15,32,33,34].

The vaccination is one of the most effective means to prevent infectious diseases. The introduction of effective vaccines against SARS-CoV-2 was expected to prevent COVID-19 cases [35]. Nowadays, the vaccines are widely available, but of limited durability in vaccine-induced immunity. The inability of subgroups of the population to be vaccinated and increased infectious and/or vaccine-insensitive variants of concerns, VOCs (Alpha, Beta, Gamma, Delta, and Omicron variants), have fueled recurring global infection waves [36,37,38,39]. The Alpha variant (B.1.1.7 lineage) was first observed in the UK and, then, soon began to spread rapidly around the world [40]. The current study showed a change in the epidemiology of the virus during the 5-month period in favor of the Alpha variant (Figure 3). The wild-type virus dominated initially, which gradually began to decline to the B.1.1.7 lineage in the following months. In March, it represented more than 96% of all identified cases. This result reflects national data where the Alpha variant dominated until May 2021 and then began to be displaced by the Delta variant [41].

The time when specimens were collected for SARS-CoV-2 testing was also the peak season for respiratory infections caused by other viruses. Therefore, a retrospective study was conducted to identify non-SARS-CoV-2 viruses in mono- and co-infections. It should be noted that the introduction of various responses to the COVID-19 pandemic, ranging from temporary room closures to the wearing of masks, social distancing, increased personal hygiene, and travel restrictions, was intended to limit the spread of SARS-CoV-2, but also affected the occurrence of other common seasonal respiratory viruses. Seasonal influenza is an acute respiratory infection caused by influenza viruses that circulate in all parts of the world. It represents a year-round disease burden. It causes illness of varying degrees of severity, which sometimes leads to hospitalization and death. As mentioned previously, before the COVID-19 era, the influenza virus was one of the most common viral etiological agents of acute RTIs [2]. During the 2019/2020 flu season, more than 4.8 million cases of influenza illness or suspected illness were reported in Poland and a total of 65 deaths were recorded [30]. In contrast, in 2020/2021, 2 million fewer reported cases and no deaths were registered [31]. Only one case of influenza virus (3.03%) was identified in this study. Our result differs from those described in other papers, where influenza virus was a higher proportion [14,15,18,42,43,44].

Rhinovirus-dependent infections occur throughout the year, with a seasonal peak in incidence in early autumn and spring. During these periods, up to 80% of colds can be associated with a documented rhinovirus infection [45]. Among viruses other than SARS-CoV-2 detected in this study, RV was the most frequently represented in mono-infections (66.67%). Our observations are similar to those of other studies [16,17,18,19,44,46].

HMPV infections can occur throughout the year, but seasonality has been described in several studies [47]. In our study, HMPV was found to be the agent of respiratory infection in 10 patients (30.30%) and was the second most commonly identified non-SARS-CoV-2 virus fallowing RV, which is consistent with other reports [16,18,44].

Before COVID-19 achieved pandemic status, reports from Wuhan, China, described a very low number of co-infections with other respiratory pathogens during SARS-CoV-2 infection [9,10,11]. At present, there are increasing reports on the integration of SARS-CoV-2 into existing circulating infection patterns [48,49]. Co-infections, compared with single infections, may lead to changes in transmission of the pathogen, progression of clinical symptoms, and the adverse effects associated with any given infection, which ultimately determines the management of infectious diseases. Co-infection (two respiratory viruses) was detected in 18 (0.95% of all tested and 1.98% positive cases) individuals, which is statistically significantly compared to mono-infections (*p* < 0.0001). As mentioned previously, a positive result for SARS-CoV-2 co-infection with other respiratory virus was found in 17 samples (94.44%). The highest rate of SARS-CoV-2 co-infections was observed with RV (76.47%), followed by HMPV and HAdV (11.76% each). In addition, one RV and EV co-infection was identified. These results are in line with data from other researchers [14,15,16,17,18,19,44,45,46,47,48,49].

A limitation of our study was that the laboratory questionnaire did not detail specific symptoms, so we cannot conclude which complaints/symptoms were most commonly reported by patients. In addition, unlike hospital-based studies, where various patient parameters are tracked, we could not follow the RTIs and their outcomes.

## 5. Conclusions

This study analyzed the viral etiological agents responsible for respiratory tract diseases. The most prevalent identified virus was SARS-CoV-2; it was statistically significantly more common in adults, regardless of gender. Although the spread of COVID-19 is a major public health concern, SARS-CoV-2 may not be the only pathogen responsible for respiratory infections. Other viruses, such as adenovirus, rhinovirus, metapneumovirus, enterovirus, and influenza, have also been detected, more frequently in mono- infections, but also in co-infections (mainly with SARS-CoV-2). It should be noted that RTI co-infections, depending on the patient’s immune system status and comorbidities, usually result in a worse prognosis for the patient.

## Figures and Tables

**Table 1:** The demographic and microbiological characteristics of the patients. The frequency of involvement of viral agents in RTIs was calculated based on the number of total patients in the sample collection/gender/age group.

Variables	No. of Samples (%)	No. of Negative Cases (%)	No. of Positive Cases (%)	SARS-CoV-2 Mono-Infection (%)	SARS-CoV-2 Co-Infection (%)	Non-SARS-CoV-2 Mono-Infection (%)	Non-SARS-CoV-2 Co-Infection (%)
**Specimen collection**	1890 (100)	980 (51.85)	910 (48.15)	859 (45.45)	17 (0.9)	33 (1.75)	1 (0.05)
**Female**	1041 (55.07)	555 (53.31)	486 (46.69)	454 (43.61)	12 (1.15)	20 (1.92)	0 (0.00)
**Male**	849 (44.92)	425 (50.06)	424 (49.94)	405 (47.70)	5 (0.59)	13 (1.53)	1 (0.12)
**Median age in years (range)**	51 (0–99)						
**0–5**	40 (2.12)	29 (72.5)	11 (27.5)	7 (17.5)	2 (5.0)	2 (5.0)	0 (0.0)
**6–18**	63 (3.33)	36 (57.14)	27 (42.86)	24 (38.10)	1 (1.59)	2 (3.17)	0 (0.0)
**19–35**	342 (18.09)	167 (48.83)	175 (51.17)	164 (47.95)	2 (0.58)	9 (2.63)	0 (0.0)
**36–64**	1023 (54.13)	505 (49.36)	518 (50.64)	491 (48.0)	10 (0.98)	17 (1.66)	0 (0.0)
**≥65**	422 (22.33)	243 (57.58)	179 (42.42)	173 (41.0)	2 (0.47)	4 (0.95)	1 (1.0)

**Table 2:** The distribution of non-SARS-CoV-2 mono-infections (RV—rhinovirus, HAdV—human adenovirus, HMPV—human metapneumovirus, EV—enterovirus, and FLUV—influenza virus) and all co-infections caused by SARS-CoV-2 and other respiratory viruses.

Age Group	Mono-Infections, *n* (Female/Male)	Co-Infections, *n* (Female/Male)
	**RV**	**SARS-CoV-2 + RV**
**Total**	22 (13/9)	13 (9/4)
**0–5**	2 (2/0)	2 (0/2)
**6–18**	2 (0/2)	0 (0/0)
**19–35**	6 (3/3)	2 (2/0)
**36–64**	11 (7/4)	7 (6/1)
**≥65**	1 (1/0)	2 (1/1)
	**HAdV**	**SARS-CoV-2 + HAdV**
**Total**	0 (0/0)	2 (2/0)
**0–5**	0 (0/0)	0 (0/0)
**6–18**	0 (0/0)	1 (1/0)
**19–35**	0 (0/0)	0 (0/0)
**36–64**	0 (0/0)	1 (1/0)
**≥65**	0 (0/0)	0 (0/0)
	**HMPV**	**SARS-CoV-2 + HMPV**
**Total**	10 (6/4)	2 (1/1)
**0–5**	0 (0/0)	0 (0/0)
**6–18**	0 (0/0)	0 (0/0)
**19–35**	3 (2/1)	0 (0/0)
**36–64**	5 (3/2)	2 (1/1)
**≥65**	2 (2/0)	0 (0/0)
	**EV**	**RV + EV**
**Total**	0 (0/0)	1 (0/1)
**≥65**	0 (0/0)	1 (0/1)
	**FLUV**	
**Total**	1 (1/0)	-
**36–64**	1 (1/0)	-

## Data Availability

All relevant data are within the manuscript.
